# TLR2-induced CD8^+^ T-cell deactivation shapes dendritic cell differentiation in the bone marrow during sepsis

**DOI:** 10.3389/fimmu.2022.945409

**Published:** 2022-09-06

**Authors:** Anne-Charlotte Antoni, Ekaterina Pylaeva, Bettina Budeus, Jadwiga Jablonska, Ludger Klein-Hitpaß, Marcel Dudda, Stefanie B. Flohé

**Affiliations:** ^1^ Department of Trauma, Hand, and Reconstructive Surgery, University Hospital Essen, University Duisburg-Essen, Essen, Germany; ^2^ Department of Otorhinolaryngology, University Hospital Essen, University Duisburg-Essen, Essen, Germany; ^3^ Institute of Cell Biology, University Hospital Essen, University Duisburg-Essen, Essen, Germany

**Keywords:** dendritic cells, differentiation, sepsis, bone marrow, T cells, immunosuppression, TLR - toll-like receptor

## Abstract

Sepsis is associated with profound immune dysregulation that increases the risk for life-threatening secondary infections: Dendritic cells (DCs) undergo functional reprogramming due to yet unknown changes during differentiation in the bone marrow (BM). In parallel, lymphopenia and exhaustion of T lymphocytes interfere with antigen-specific adaptive immunity. We hypothesized that there exists a link between T cells and the modulation of DC differentiation in the BM during murine polymicrobial sepsis. Sepsis was induced by cecal ligation and puncture (CLP), a model for human bacterial sepsis. At different time points after CLP, the BM and spleen were analyzed in terms of T-cell subpopulations, activation, and Interferon (IFN)-γ synthesis as well as the number of pre-DCs. BM-derived DCs were generated *in vitro*. We observed that naïve and virtual memory CD8^+^ T cells, but not CD4^+^ T cells, were activated in an antigen-independent manner and accumulated in the BM early after CLP, whereas lymphopenia was evident in the spleen. The number of pre-DCs strongly declined during acute sepsis in the BM and almost recovered by day 4 after CLP, which required the presence of CD8^+^ T cells. Adoptive transfer experiments and *in vitro* studies with purified T cells revealed that Toll-like receptor 2 (TLR2) signaling in CD8^+^ T cells suppressed their capacity to secrete IFN-γ and was sufficient to change the transcriptome of the BM during sepsis. Moreover, the diminished IFN-γ production of CD8^+^ T cells favored the differentiation of DCs with increased production of the immune-activating cytokine Interleukin (IL)-12. These data identify a novel role of CD8^+^ T cells in the BM during sepsis as they sense TLR2 ligands and control the number and function of *de novo* differentiating DCs.

## Introduction

Sepsis is defined as “life-threatening organ dysfunction caused by a dysregulated host response to infection” (1) and may be caused by bacteria, fungi, and viruses that rapidly trigger the innate immune system (2). The consequence is a systemic hyperinflammation that may cause life-threatening multi-organ failure. Owing to the improvement in intensive care medicine during the last decades, most patients survive the acute hyperinflammation but then face an enhanced risk for detrimental nosocomial infections and persistent organ failure (3, 4). Despite major effort in medical research, the underlying mechanisms of the imbalance between “hyperinflammation” on the one hand and “immunosuppression” (indicative of the enhanced susceptibility to secondary infections) on the other hand are only incompletely understood (5, 6).

Innate immune cells such as neutrophils, monocytes, dendritic cells (DCs), and NK cells are stimulated by invading pathogens and provide an early unspecific response to the infection. With some delay, T lymphocytes are activated upon recognition of their cognate antigen in conjunction with human leukocyte antigen (HLA) molecules on antigen-presenting cells. The antigen-specific activation of naïve T (T_N_) cells finally results in the formation of T-cell memory that protects the host from recurrent infection with the same pathogen. There is increasing evidence that central memory T (T_CM_) cells, a subpopulation of antigen-experienced T cells, moreover may be activated by innate cytokines such as IL-12, IL-15, and IL-18 independent of their cognate antigen (7). Similarly, virtual memory T (T_VM_) cells, which phenotypically resemble T_CM_ but have not been exposed to their antigen yet, rapidly respond to cytokine stimulation with the synthesis of IFN-γ (often termed “bystander activation”) and thereby may contribute to the early defense against infection (8, 9). T_VM_ can be distinguished from true memory T cells according to their low expression of CD49d but high expression of the shared β chain of the IL-2 and IL-15 receptor (CD122) (10).

The immune dysregulation during sepsis affects both the innate and the adaptive immune system. Human monocytes express reduced levels of HLA-DR and fail to release pro-inflammatory cytokines in response to lipopolysaccharide (LPS). Because antigen-specific T-cell responses are crucial for effective elimination of diverse pathogens, the restoration of HLA-DR expression and cytokine secretion by monocytes has been considered as a promising therapeutic approach to reduce the risk for secondary infections during sepsis. However, neither the administration of granulocyte-macrophage colony-stimulating factor (GM-CSF) nor therapy with IFN-γ has presented the expected clear benefit in clinical studies so far, although these approaches successfully restored HLA-DR expression and cytokine secretion by monocytes (5, 11). These findings create some doubts on the relevance of HLA-DR expression during sepsis.

Regarding the adaptive immune system, a large number of T lymphocytes rapidly undergo apoptosis early during sepsis, which results in lymphopenia that is associated with disease severity (12). The remaining T cells are impaired in IFN-γ synthesis upon antigen-dependent and antigen-independent activation for so far unclear reason and show signs of T-cell exhaustion (13, 14). Consequences of the inhibition of the antigen-specific T-cell response during murine sepsis are the reactivation of Lymphocytic choriomeningitis virus infection and impaired immunity to *Listeria monocytogenes* (15, 16). An important so far unconsidered issue is that nosocomial infections in patients with sepsis are frequently caused by rapidly dividing bacteria such as *E. coli*, *Staphylococcus aureus*, and *Pseudomonas aeruginosa* (17). The elimination of such pathogens mainly relies on innate immune cells and, thus, cannot be explained by impaired antigen-specific T-cell responses.

DCs are professional antigen-presenting cells and reside in lymphoid and non-lymphoid tissues at low numbers. DCs sense invading pathogens by diverse pathogen recognition receptors such as Toll-like receptors (TLRs). Owing to their ability to secrete numerous cytokines such as IL-12 and to express high levels of MHC molecules, DCs may interact with innate and adaptive immune cells and thereby orchestrate protective immunity (18). There exist two subsets of DCs, namely, DC1 and DC2, that may exert different function with regard to cytokine secretion and T-cell activation (19).

DCs differentiate from distinct progenitor cells in the bone marrow (BM). At the stage of pre-DCs, commitment toward pre-DC1 or pre-DC2 occurs (20). Pre-DCs are released into the circulation and migrate into peripheral tissues where they undergo final differentiation to conventional DCs (21). DCs may additionally differentiate from monocytes (19). Importantly, DCs may phenotypically belong to the same subset but differ in function depending on the tissue they are located. This led to the current concept that progenitor cells in the BM follow a common program to differentiate to pre-DCs but that the local microenvironment during final differentiation to DCs in the periphery dictates a tissue-specific function (19, 22).

The function of DCs is severely disturbed during sepsis as they rapidly lose their capacity to secrete the immune-activating cytokine IL-12 in response to microbial stimuli and to induce antigen-specific CD4^+^ and CD8^+^ T-cell responses (23, 24). DCs from septic hosts secrete enhanced levels of the regulatory cytokine IL-10 that dampens the differentiation of IFN-γ–producing T helper type 1 (Th1) cells and the activation of NK cells (23). In addition, the number of DCs is reduced in the secondary lymphoid organs presumably due to apoptosis (24–26). This dysfunction of DCs contributes to the increased susceptibility of the host to secondary infection (23, 24). There is evidence that the functional reprogramming of DCs during sepsis is mediated by a modulation of DC differentiation in the BM rather than on signals that they receive in the local tissue in the periphery (23).

The current knowledge on T-cell function during sepsis mainly relies on the examination of the peripheral blood and of the secondary lymphoid organs. In contrast, limited information exists on T cells in the BM. The BM is a preferential site for memory T cells during homeostasis and contributes to the recovery of the antigen-experienced memory CD4^+^ T cell pool in the periphery in a long term after sepsis (27). The simultaneous appearance of impaired T-cell function and DC dysregulation led us to the hypothesis that T cells are involved in the modulation of DC differentiation. Here, we show that, early during sepsis, bystander-activated CD8^+^ T cells accumulate in the BM, are impaired in IFN-γ synthesis due to TLR2 signaling, and control the number and the function of differentiating DCs.

## Material and methods

### Animals

Female BALB/cAnNRj mice (10–14 weeks old, 20–23 g) were obtained from Janvier Labs, Saint Berthevin Cedex, France, and served as wildtype (WT) mice. TLR2^−/−^ mice, IFN-γ^−/−^, CD45.1 congenic, and DO11.10 transgenic mice on BALB/c background were bred at the local animal facility of the University Hospital Essen. All mice were kept under specific pathogen–free conditions and had access to standard rodent food and water ad libitum.

### Induction of polymicrobial sepsis and applications

Polymicrobial sepsis was induced through cecal ligation and puncture (CLP) as described previously (23, 28). Briefly, mice were anesthetized with ketamine (100 mg/kg) and xylazine (10 mg/kg) and underwent a midline laparotomy. The cecum was exposed, ligated by 50%, and punctured once with a 27-gauge needle to extrude a small amount of cecum content. Thereafter, the cecum was replaced, 1 ml of warm sterile saline was injected to resuscitate the mice, and the incision was closed in two layers. Finally, Temgesic^®^ (10 g/kg body weight; Buprenorphine; Indivior, Mannheim, Germany) was administered for pain relief. Sham mice underwent the same surgery except for the ligation and puncture of the cecum. Under these conditions, CLP mice showed symptoms of illness within 24 h after surgery in form of weight loss, rough fur, and reduced mobility. The mortality rate was less than 20% within 4 days after surgery. The animals did not receive antibiotics. For CD8^+^ T cell depletion, mice were treated with an intraperitoneal (i.p.) injection of antibodies against CD8β (InVivoMAb CD8β, BioXCell, Lebanon, USA; 100 µg per mouse) or isotype control antibodies 3 days before surgery. Previous experiments have shown that this treatment regimen caused a systemic depletion of CD8^+^ T cells for 6–8 days after administration. The application of these antibodies does not target the DC1 subpopulation that expresses the CD8α chain but not the CD8β chain.

For T-cell transfer, CD8^+^ T cells were isolated from the spleen of WT or TLR2^−/−^ mice using the CD8^+^ T Cell Isolation Kit (Miltenyi Biotec, Bergisch Gladbach, Germany) according to the manufacturer’s protocol. The purity of isolated CD8^+^ T cells was more than 95% as determined by flow cytometry. One million cells were injected intravenously (i.v.) into the tail vein of the recipient animal immediately before surgery.

### Preparation and culture of cells

Very low–endotoxin RPMI 1640 (Biochrom, Berlin, Germany) supplemented with 10% fetal calf serum (Biochrom), 10 mM HEPES (Biochrom), 2 mM glutamine, penicillin (0.06 mg/ml), gentamicin (0.02 mg/ml), and 0.05 mM 2-mercapthoethanol (all from Sigma-Aldrich, Taufkirchen, Germany) was used as culture medium (CM). To isolate total spleen cells (TSC), the spleen was removed and digested with collagenase as described previously (26). Red Blood Cell Lysing Buffer (Sigma-Aldrich) was used to lyse erythrocytes.

BM cells (BMC) were isolated as described before (23). Briefly, the cells were flushed out from tibiae and femurs using CM, gently resuspended, and filtered through a 30-µm filter. Erythrocytes were lysed using the Red Blood Cell Lysing Buffer. For quantification of intracellular cytokine production in T cells, 5 × 10^5^ BMC/well were cultured in 96-well plates and stimulated with phorbol myristate acetate (PMA) (10 ng/ml; Sigma-Aldrich) and Ionomycin (1 µg/ml; Sigma-Aldrich). Brefeldin A (GolgiPlug, BD Biosciences, Heidelberg, Germany) was added during the last 5 h of culture. For the generation of supernatants, purified CD8^+^ splenic T cells from naïve WT mice were cultured in the absence or presence of a cytokine cocktail consisting of recombinant mIL-12 (20 ng/ml; R&D Systems, Wiesbaden, Germany), recombinant mIL-18 (20 ng/ml; MBL Co., Ltd., Naka-ku Nagoya, Japan), and IL-15 (10 ng/ml; PeproTech, Rocky Hill, USA). After 24 h, the supernatants were harvested. Where indicated, cells were pretreated with different concentrations of P_3_CSK_4_ (*In vivo* Gen, San Diego, USA) for 24 h before stimulation with PMA/Ionomycin or with the cytokine cocktail.

To generate BM-derived DCs (BMDCs), 2 × 10^5^/ml BMC were cultured in CM containing recombinant murine granulocyte-macrophage colony-stimulating factor (GM-CSF) (15 ng/ml; PromoKine, PromoCell, Heidelberg, DE) for 8 days (29). Non-adherent BMDCs were harvested and stimulated with LPS (100 ng/ml; *E. coli* 026:B6; Sigma-Aldrich, Taufkirchen, Germany) or with CpG (5 µg/ml; ODN1668, *In vivo* Gen) for 18–20 h. Supernatants were stored at −20°C for further analyses.

### Whole-blood analysis

Blood was drawn and transferred to a 1.3-ml K3E Membrane Micro tube (Sarstedt, Nümbrecht, Germany), which includes 1.6 mg of EDTA/ml, and/or into a 1.2-ml S-Monovette for serum preparation (Sarstedt) including a clot activator. After at least 30 min, the S-Monovettes were centrifuged (2,000*g*, 10 min at room temperature), and the serum was aspirated and stored at −20°C until further use.

### Analyses of sera and supernatants

The ELISA DuoSet Mouse IL-10, IL-12p70, IL-6, IFN-γ, and TNFα (R&D Systems) were used according to the manufacturer’s protocol to determine the cytokine levels. The EDTA blood was analyzed for blood urea nitrogen (BUN) and creatine phosphokinase (CPK) levels using the Spotchem II (Arkray, Kyōto, Japan) or for their leukocyte distribution using the Vet abc™ (Scil animal care, Viernheim, Germany).

### Flow cytometry

Surface staining was performed using various combinations of fluorochrome-labeled antibodies against B220 (clone RA3-6B2), CD3 (clone 17A2, 145-2C11), CD4 (clone RM4-5), CD8 (clone 53-6.7), CD11b (clone M1/70), CD11c (clone N418), CD40 (clone HM40-3), CD44 (clone IM7), CD45.1 (clone A20), CD49d (clone 9C10), CD62L (clone MEL-14), CD69 (clone H1.2F3), CD86 (clone GL1), CD122 (clone TM-β1), CD135 (clone A2F10), DO11.10 TCR (clone KJ1-26), Fixable Viability Dye (FvD), I-A/I-E (clone M5/114.15.2), Ly6C (clone AL-21), PD1 (clone 29F.1A12), and Siglec-H (clone 551) for 15 min in the dark at 4°C. Appropriate isotype controls were used to define the threshold of negative versus positive staining. Thereafter, the cells were washed using Cell Wash (BD Biosciences). In the case of intracellular staining for IFN-γ, the cells were incubated with 150 µl of Fixation/Permeabilization solution (BD) for 20 min at room temperature in the dark. After washing with BD Perm/Wash™ Buffer, the cells were incubated for 15 min at 4°C with an IFN-γ antibody (clone XMG1.2) or with the respective isotype control antibody. Finally, the cells were washed and resuspended in CellWash and analyzed within 1 day. All antibodies were purchased from BD Biosciences, eBioscience (Frankfurt am Main, Germany), or BioLegend (San Diego, USA). Data were acquired using a FACSCanto II (BD Biosciences) or Cytoflex (Beckman Coulter, Pasadena, USA). Data analysis was performed using FACSDiva Software or NovoExpress (ACEA Biosciences, San Diego, CA, USA). Separate analyses with FvD indicated that freshly isolated cells contained less than 10% dead cells that could be excluded according to their characteristic FSC^lo^/SSC^lo^ pattern. In the case of intracellular cytokine staining of cultured cells, FvD was added to the panel and FvD^+^ cells, indicating dead cells, were excluded.

### RNA array

RNA was isolated using the RNeasy Mini Kit (Qiagen, Düsseldorf, Germany). Gene expression profiling (GEP) was performed using high-density oligonucleotide arrays (Clariom S mouse, Affymetrix, Santa Clara, CA, USA). Target preparation was performed according to the Affymetrix WTPlus Expression Protocol with approximately 100 ng of total RNA for the analysis on the Affymetrix Clariom S mouse array. Hybridization, washing, and staining of the arrays were done according to the Affymetrix recommendations on the GC Scanner 3000 with G7 update.

To identify differentially expressed genes, statistical analyses were performed in which the experimental samples were compared to the respective baseline samples by using the RMA signal summarization method and ANOVA test implemented in the Affymetrix Transcriptome Analysis Suite (Folder 3_TAC_analysis).

Gene set enrichment analysis (GSEA) was performed with RMA signals calculated with the Affymetrix Expression Console, GSEA software version 4.1, and MSigDB geneset collection v7.4 using default settings and gene set permutation. Pathways showing a false discovery rate (FDR) <0.25 and a normalized enrichment score (NES) > 1.5 were identified.

### Statistical analyses

The data are shown as individual values with median and interquartile range or as mean ± SD as indicated. Differences between two groups were tested using the non-parametric Mann–Whitney U-test or the unpaired Student *t*-test. Correlation was analyzed using the Spearman rank correlation test. A p-value ≤ 0.05 was considered statistically significant. Statistical analyses and layout of the graphs were performed using GraphPad Prism versions 5 and 8.

## Results

### Sepsis changes the pre-DC subset distribution in the BM

**Figure 1:**
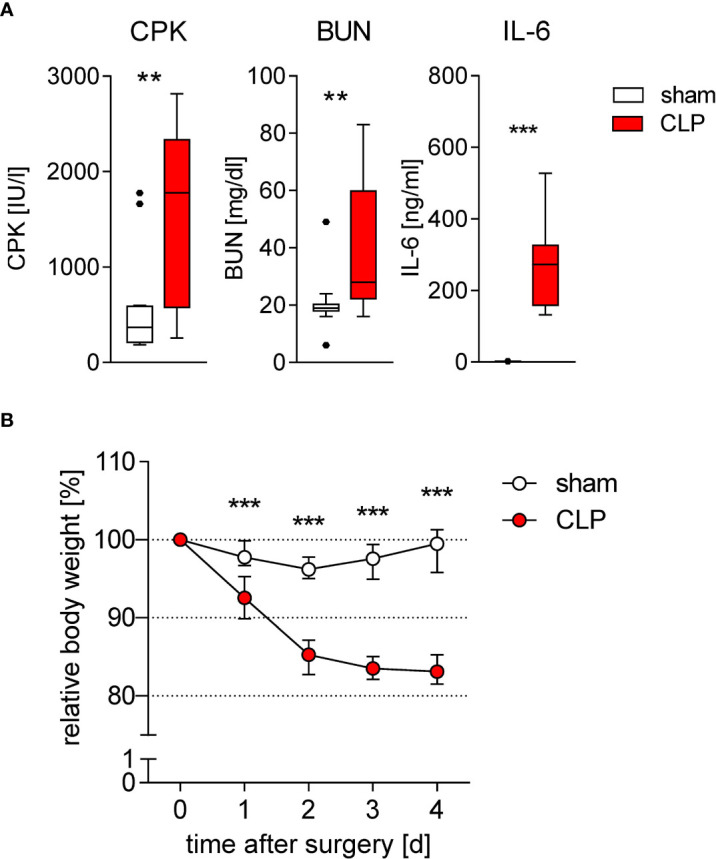
Induction of inflammation, organ damage, and weight loss during sublethal sepsis. WT mice underwent sham or CLP surgery. **(A)** Concentration of creatine phosphokinase (CPK) as marker for muscle cell degradation, blood urea nitrogen (BUN) as marker for kidney damage, and IL-6 as marker for inflammation in the serum 24 h after surgery (each n = 10–15 mice per group). **(B)** Time course of the body weight after surgery. Data were normalized to the individual body weight before surgery (set as 100%). Data show median and interquartile range of n = 12 mice per group per time point. Significant differences between sham and CLP mice were tested using Mann–Whitney *U*-test. **p ≤ 0.01; ***p ≤ 0.001.

To get further insight into the functional reprogramming of DCs during sepsis, we used a moderate degree of CLP that caused a mortality rate of 10%–20% (23). As known for severe sepsis, increased levels of CPK, BUN, and IL-6 were detected in the sera of mice, indicating skeletal muscle degradation, kidney injury, and systemic inflammation, respectively, during the acute phase of moderate sepsis (**Figure 1A**). Furthermore, sepsis induced weight loss during the initial 4 days after CLP (**Figure 1B**). In line with previous reports (23–25, 30), the reduction in the total number of conventional DCs in the spleen (**Supplementary Figures 1A, B**) was mainly based on a loss of the CD4^+^ DC2 subset that was evident 24 h after induction of sepsis and that was maintained at least 4 days after CLP (**Supplementary Figure 1C**).

**Figure 2:**
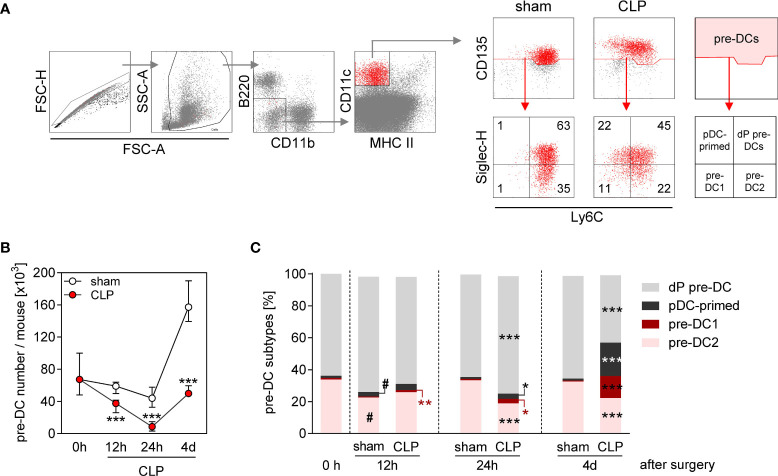
Decline in pre-DC number and altered pre-DC commitment in the BM during sepsis. WT mice underwent sham or CLP surgery and BM cells were isolated at indicated time points thereafter. **(A)** Gating strategy of pre-DCs (B220^−^CD11b^−^CD11c^+^MHCII^−^CD135^+^). Pre-DC subsets were distinguished according to the expression of Ly6C and Siglec-H: double-positive pre-DCs (dP pre-DCs; Ly6c^+^Siglec-H^+^), pDC-primed pre-DCs (Ly6c^-^Siglec-H^+^), pre-DC1 (Ly6c^-^Siglec-H^−^), and pre-DC2 (Ly6c^+^Siglec-H^−^). Representative dot plots of one sham and one CLP mouse (4 days after surgery) are shown. Numbers indicate the percentage in the respective quadrant. **(B)** Absolute number of pre-DCs per mouse BM. **(C)** Distribution of pre-DCs on dP pre-DCs, pDC-primed pre-DCs, pre-DC1, and pre-DC2. Data show the median with interquartile range **(B)** or median **(C)** of n = 4 (0 h; equivalent to naïve mice) or n = 7–14 mice per group. Significant differences were tested using Mann–Whitney U-test. *p ≤ 0.05; **p ≤ 0.01; ***p ≤ 0.001 between sham and CLP mice. ^#^p ≤ 0.05 versus “0 h”. pDC, plasmacytoid DC.

Splenic DCs are replaced by “new” DCs with a half-life time of 2–3 days (31). We asked whether the prolonged reduction in the conventional DC number during sepsis was associated with an alteration in DC precursor cells in the BM. Total pre-DCs consist of pre-DC1, pre-DC2, plasmacytoid DC-committed pre-pDCs, and uncommitted pre-DCs that all can be distinguished according to their expression of Ly6C and Siglec-H (20) (for gating, see **Figure 2A**). The number of pre-DCs strongly declined within the first 24 h after CLP and almost recovered by 4 days after CLP (**Figures 2A, B**). Notably, there was a predominant increase of the populations of pre-DC1 and plasmacytoid DC-primed pre-DC precursors at the later time point (**Figure 2C** and **Supplementary Figure 2**). In contrast, sham surgery did not affect the number of pre-DCs during the first 24 h but caused an expansion of the pre-DC population thereafter without bias toward a distinct subset (**Figure 2C**). Thus, the population of pre-DCs largely disappears from BM during acute sepsis and thereafter recovers with an altered distribution of the pre-DC subsets.

### Activated CD8^+^ T cells accumulate in the BM during sepsis

**Figure 3:**
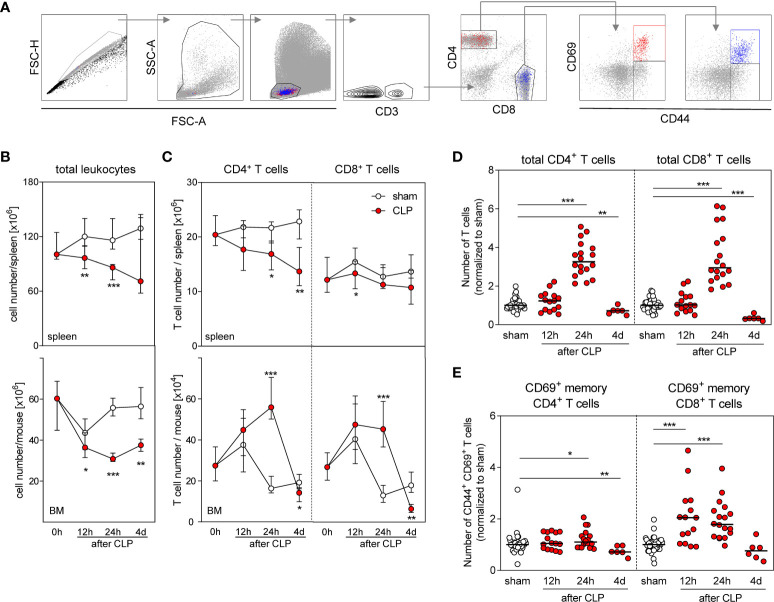
Accumulation and activation of CD8^+^ T cells in the BM during sepsis. Sepsis was induced in WT mice and BM cells as well as total spleen cells were isolated at the indicated time points after CLP or sham surgery. T cell number and expression of CD69 were determined. **(A)** Gating strategy. Representative dot plots of BM cells 24 h after CLP are shown. **(B)** Number of total spleen cells (top) and BM cells (bottom). **(C)** Number of total CD4^+^ and CD8^+^ T cells in the spleen (top) and BM (bottom). **(D)** Number of CD4^+^ and CD8^+^ T cells in the BM normalized to the median of sham mice that was set as 1. **(E)** Percentage of CD69^+^ cell among memory CD44^+^ CD4^+^ and CD8^+^ T cells normalized to the median of sham mice that was set as 1. Data show median/interquartile range **(B, C)** or median **(D, E)** of n = 7–15 (spleen) or n = 10–16 (BM) mice per group. Mann–Whitney *U*-test was used to test for statistically significant differences. *p ≤ 0.05; **p ≤ 0.01; ***p ≤ 0.001.

To evaluate a potential involvement of T lymphocytes in the modulation of DC differentiation, we analyzed the T cell compartment in the BM and in the spleen (the gating strategies are shown in **Figure 3A**). As expected, the number of leukocytes in the spleen declined after CLP mainly as result of a loss of CD4^+^ T cells (**Figures 3B, C**). The number of CD8^+^ T cells in the spleen did not change considerably (**Figure 3C**). Similarly, the number of total BMCs declined after CLP (**Figure 3B**). Remarkably, CD4^+^ and CD8^+^ T cells accumulated in the BM after CLP (**Figure 3C**) and led to a three-fold increased T cell number (in comparison with sham treatment) by 24 h after sepsis induction that is in contrast to lymphopenia in the spleen (**Figure 3D**). A fraction of both CD4^+^ and CD8^+^ T cells expressed the early activation marker CD69 on the surface that was mainly attributed to CD44^+^ cells (**Figure 3A**). The number of such CD69^+^CD44^+^ cells among CD8^+^ T cells increased by two-fold 12 and 24 h after CLP before it declined to the number found in the BM of sham mice (**Figure 3E**). The number of CD69^+^CD44^+^ cells among CD4^+^ T cells did not increase considerably after CLP (**Figure 3E**). Although the total number of CD8^+^ T cells transiently declined in the spleen, the number of CD69^+^CD8^+^CD44^+^ T cells increased (**Supplementary Figure 3**).

**Figure 4:**
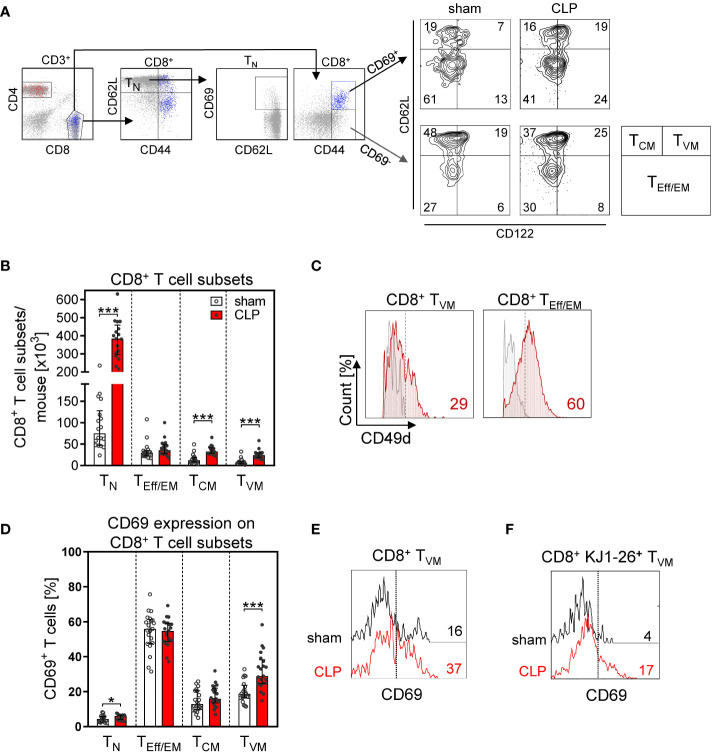
CD8^+^ T-cell subset composition and antigen-independent CD8^+^ T cell activation in the BM after CLP. Sepsis or sham surgery was induced in WT mice and BM cells were isolated 24 h later. The expression of CD69 on diverse CD8^+^ T-cell subsets was determined by flow cytometry. **(A)** Gating strategy of naïve (T_N_), effector/effector memory (T_Eff/EM_), central memory (T_CM_), and virtual memory (T_VM_) CD8^+^ T cells. Representative contour plots of one sham and one CLP mouse are shown. Numbers indicate the percentage of cells in the respective quadrant. **(B)** Cell count of CD8^+^ T-cell subsets from individual mice. **(C)** Expression of CD49d on BM CD8^+^ T_VM_ cells in comparison with splenic CD8^+^ T_Eff/EM_ cells as positive control. Numbers indicate the percentage of positive cells according to fluorescence minus one (FMO) indicated as gray line. **(D)** CD69 expression on CD8^+^ T cell subsets from individual mice. Horizontal lines indicate the median with interquartile range of n = 17–21 mice per group. **(E)** Histogram of CD69 expression of gated CD8^+^ T_VM_ from one representative sham and one CLP mouse. The dashed line indicates the threshold for positive staining according to the isotype control. **(F)** CD8^+^ T cells from DO11.10 mice were adoptively transferred into WT mice prior to CLP or sham surgery. Transferred ovalbumin-specific cells were identified as KJ1-26^+^ cells. The histogram depicts the expression of CD69 on gated KJ1-26^+^ T_VM_ cells from one representative sham and one CLP mouse. Statistically significant differences were tested using the Mann–Whitney *U*-test. *p < 0.05; ***p < 0.001.

More detailed analyses of the CD8^+^ T cell subsets revealed that the increase in the total CD8^+^ T cell number in the BM after CLP was largely mediated by a prominent expansion of the T_N_ cell and T_CM_ population (**Figures 4A, B**). In contrast, the population of effector/effector memory (T_Eff/EM_) cells remained unchanged. A population of CD62L^+^CD44^hi^CD122^hi^CD49d^lo^ CD8^+^ T_VM_ cells expanded by three- to four-fold after CLP (**Figures 4B, C**) and moreover displayed an enhanced expression of CD69 (**Figures 4D, E**). To address whether these T cells were bystander-activated CD8^+^ T_VM_ cells, CD8^+^ T cells from DO11.10 mice that express a T-cell receptor (TCR) specific for an ovalbumin peptide/MHC II complex were adoptively transferred before induction of sepsis. Because of the MHC restriction, CD8 T cells cannot be activated by this peptide/MHC II complex. Nevertheless, the transferred CD8^+^ T_VM_ cells from DO11.10 mice displayed an increased expression of CD69 after CLP (**Figure 4F**) similar to transferred CD8^+^ T_VM_ from WT mice (**Supplementary Figure 4**), which points to an antigen-independent activation.

To evaluate whether an increased proliferation might have contributed to the expansion of CD8^+^ T-cell subpopulations in the BM, we transferred carboxyfluorescein succinimidyl ester (CFSE)-labeled CD8^+^ T cells before induction of sepsis or sham surgery. The majority of CD8^+^ T_N_ and T_VM_ cells had undergone cell division as indicated by the diminished content of CFSE. However, the proliferative activity of CD8^+^ T cells did not differ between sham and CLP mice (**Supplementary Figure 5**). Thus, CD8^+^ T_VM_ cells accumulate in the BM during the acute phase of sepsis and display an activated phenotype.

### CD8^+^ T cells support the generation of pre-DCs and immune-activating DCs

**Figure 5:**
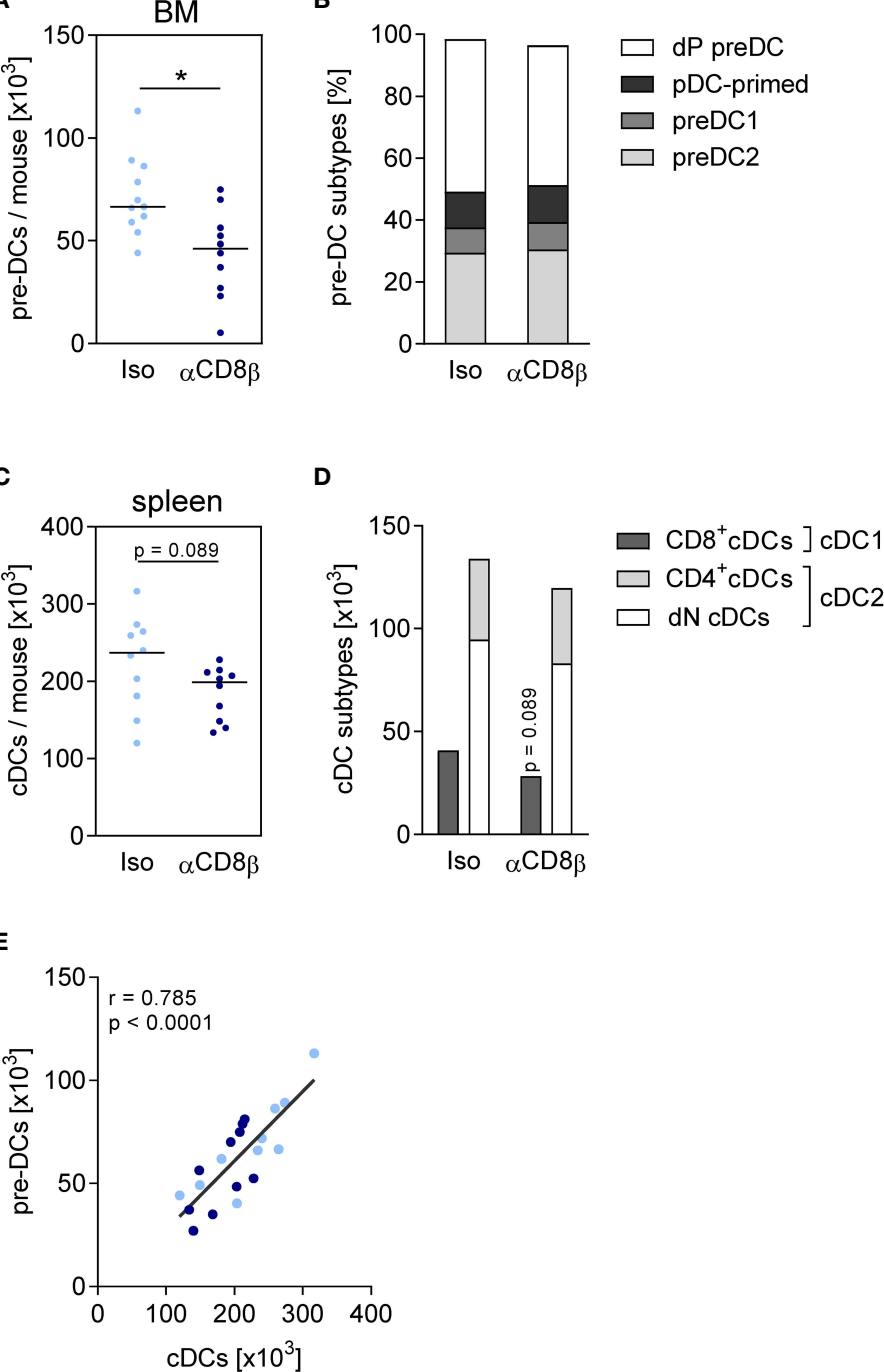
CD8^+^ T cells increase the size of the pre-DC population in the BM. Sepsis was induced in WT mice after CD8^+^ T cell depletion (αCD8β) or control treatment (iso). Four days after CLP or sham surgery, pre-DCs in the BM and conventional DCs (cDCs) in the spleen were analyzed by flow cytometry. **(A)** Total number and **(B)** subset composition of pre-DCs. **(C)** Total number and **(D)** subset composition of cDCs. Data show individual values and/or the median of n = 10–14 mice per group. The Mann–Whitney U-test was used for statistical analysis. **(E)** Spearman correlation of pre-DC number in the BM and splenic cDC number. *p ≤ 0.05. dP, double positive; pDC, plasmacytoid; dN, double negative.

To address the question whether CD8^+^ T cells that accumulated in the BM during sepsis had an impact on the differentiation of DCs, CD8 T cells were depleted before CLP using anti-CD8β antibodies (the efficacy of depletion is shown in **Supplementary Figure 6**). The depletion of CD8^+^ T cells did not change the characteristics of sepsis in terms of weight loss (**Supplementary Figure 7**), neutrophilia, and lymphopenia on day 4 after surgery (**Supplementary Figure 8**), suggesting that CD8^+^ T cells did not play a major role in the acute phase of sepsis. The depletion of CD8^+^ T cells caused a decline in the number of pre-DCs in the BM that could not be ascribed to the loss of a specific pre-DC subset (**Figures 5A, B**). There was a tendency for a reduced number of conventional DCs in the spleen after depletion of CD8^+^ T cells without effect on the frequency of DC1 and DC2 subpopulations (**Figures 5C, D**). The number of pre-DCs in the BM strongly correlated with the number of splenic conventional DCs (**Figure 5E**).

**Figure 6:**
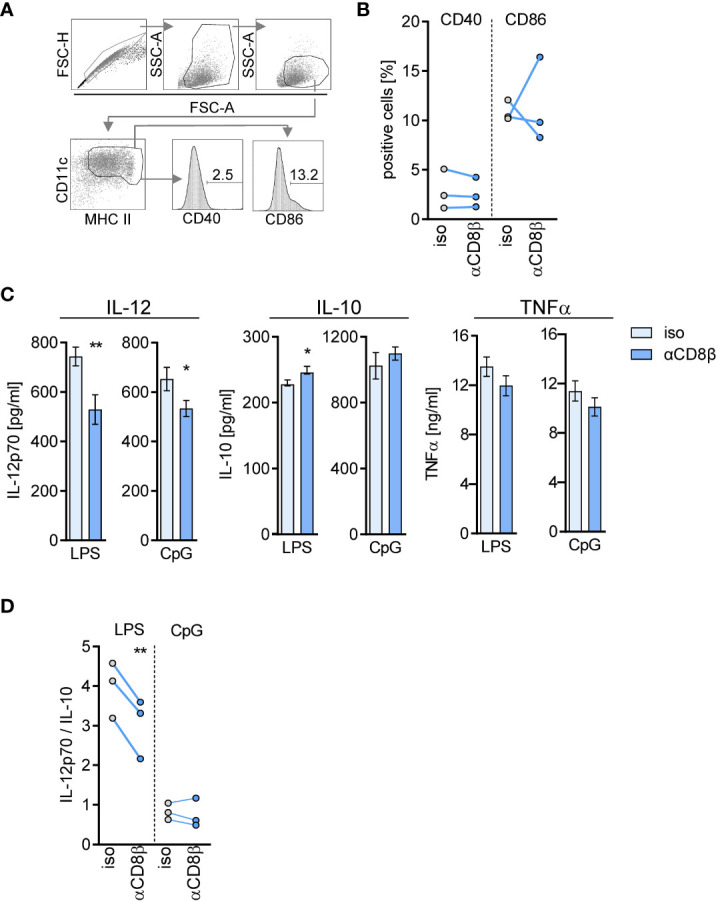
CD8^+^ T cells in the BM instruct differentiating DCs for cytokine secretion during sepsis. WT mice (n = 3–4 mice per group) were treated with anti-CD8β (αCD8β) or with the isotype control (iso) antibodies before induction of sepsis. Four days after CLP, BM cells were isolated and pooled to generate BM-derived DCs (BMDC). **(A)** Gating strategy for the analysis of CD40 and CD86 expression on CD11c^+^MHC II^+^ BMDC. **(B)** Cumulative data of CD40 and CD86 expression of three independent experiments. **(C)** BMDCs were stimulated with LPS or CpG and the release of IL-12p70, IL-10, and TNFα into the supernatant was quantified. Data show the mean ± SD of triplicate cultures of one representative experiment. Unstimulated BMDC did not secrete these cytokines (not shown). Statistically significant differences were tested using unpaired Student t-test. **(D)** Cumulative data of the ratio of IL-12/IL-10 secretion of three independent experiments. Statistically significant differences were tested using paired Student t-test. *p ≤ 0.05; **p ≤ 0.01.

Using the same experimental approach of CD8^+^ T cell depletion, we evaluated the impact of CD8^+^ T cells on the phenotype and cytokine secretion of differentiating DCs from septic mice. BMDCs were generated from BM *in vitro* and stimulated with LPS and CpG as agonists of TLR4 and TLR9, respectively. The depletion of CD8^+^ T cells before CLP did not significantly modulate the expression of the costimulatory molecules CD40 and CD86 on CD11c^+^MHCII^+^ BMDC (**Figures 6A, B**) but led to a decreased release of IL-12. The synthesis of IL-10 did not change considerably (**Figure 6C**). Consequently, the ratio of IL-12/IL-10 as a measure for the balance between immune-activating and regulatory activity decreased upon stimulation with LPS but not in response to CpG (**Figure 6D**). The release of TNF-α did not differ between BMDC of both groups (**Figure 6C**). Thus, CD8^+^ T cells support the generation of pre-DCs in the BM during sepsis and favor the differentiation of BMDC with increased immune-activating cytokine secretion.

### CD8^+^ T cells decrease their IFN-γ production in a TLR2-dependent manner

**Figure 7:**
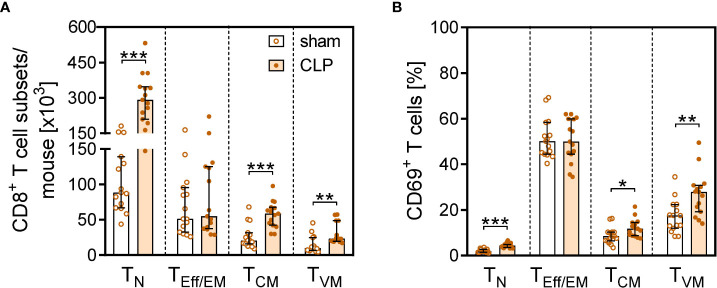
CD8^+^ T cell subset composition and activation after CLP in TLR2^−/−^ mice. Sepsis was induced in TLR2^−/−^ mice. After 24 h, CD8^+^ T cell subsets in the BM and their expression of CD69 were analyzed by flow cytometry. **(A)** Number of CD8^+^ T cells of each subset. **(B)** Percentage of CD69^+^ cells within each subset. Data show median with interquartile range and individual values of n = 15 mice per group. Mann–Whitney U-test was performed for statistical analysis. *p ≤ 0.05; **p ≤ 0.01; ***p ≤ 0.001. T_N_, naïve; T_Eff/EM_, effector/effector memory; T_CM_, central memory; T_VM_, virtual memory CD8^+^ T cells.

TLR2 serves as a costimulatory receptor on CD8^+^ T cells and thereby lowers the threshold for T-cell activation (32–35). We speculated that TLR2 ligands that circulate during polymicrobial sepsis might contribute to the activation of CD8^+^ T cells and therefore investigated the phenotype of CD8^+^ T cells in TLR2^−/−^ mice. CLP induced the expansion of the T_N_, T_CM_, and T_VM_ subpopulations (**Figure 7A**) and an increased expression of CD69 on T_N_ and T_VM_ cells (**Figure 7B**) in the BM of TLR2^−/−^ mice in a similar pattern as observed for WT mice (**Figures 4B, D**), which argues against a role of TLR2 in the activation of CD8 T cells during sepsis.

**Figure 8:**
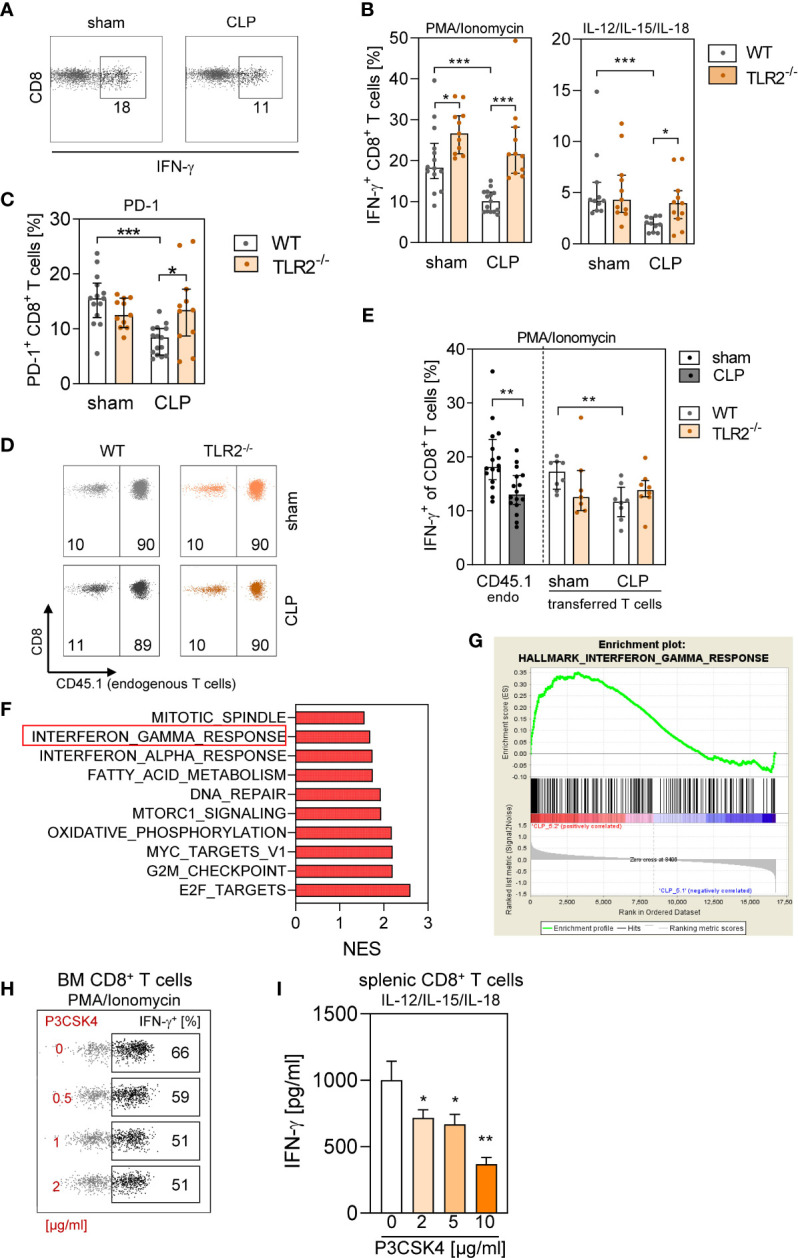
Signaling through TLR2 in CD8^+^ T cells early during sepsis interferes with IFN-γ synthesis. **(A–C)** Sepsis or sham surgery was induced in WT or TLR2^−/−^ mice. BM cells were isolated 24 h later and stimulated with PMA/Ionomycin or with IL-12/IL-15/IL-18. **(A)** Representative dot plots for IFN-γ production of gated CD8^+^ T cells from WT mice after stimulation with PMA/Ionomycin. Numbers indicate the percentage of positive cells. **(B)** Cumulative data for IFN-γ synthesis by CD8^+^ T cells for WT and TLR2^−/−^ mice (10–11 per group). **(C)** PD-1 expression on CD8^+^ T cells from WT (n = 13–14 per group) and TLR2^−/−^ (n = 11 per group) mice *ex vivo*. **(D–F)** CD45.1^+^ congenic mice received CD8^+^ T cells from WT (CD45.1^−^) or from TLR2^−/−^ (CD45.1^−^) mice before CLP or sham surgery and BM cells were isolated 24 h later (n = 7–8 mice per group). **(D)** Total CD8^+^ T cells were gated, and endogenous (endo) CD45.1^+^ and transferred CD45.1^−^ CD8^+^ T cells were discriminated. Numbers indicate the percentage of endogenous and transferred T cells among total CD8^+^ T cells. **(E)** After stimulation with PMA/Ionomycin, intracellular IFN-γ synthesis in endogenous CD45.1^+^ CD8^+^ T cells of the recipient mice and in CD45.1^−^ adoptively transferred CD8^+^ T cells was examined. Because the genotype of the adoptively transferred T cells did not change the frequency of endogenous IFN-γ^+^ CD8^+^ T cells, the data for all sham and CLP recipient mice were pooled. Bar graphs show median and interquartile range as well as individual values. Statistical differences were tested using Mann–Whitney *U*-test. **(F, G)** RNA was isolated from BM cells of CLP mice that had received WT or TLR2^−/−^ CD8^+^ T cells and used for transcriptome profiling and gene set enrichment analysis. **(F)** Normalized enrichment score (NES) of the 10 most differently expressed pathways (all with a false discovery rate <0.25). **(G)** Enrichment plot of the Hallmark pathway “Interferon gamma response” indicating genes enriched after transfer of TLR2^−/−^ CD8^+^ T cells (“CLP5.2”) in comparison with transfer of WT CD8^+^ T cells. **(H)** BM cells from naïve WT mice were exposed to various concentrations of P3CSK4 and stimulated with PMA/Ionomycin 18 h thereafter. The frequency of IFN-γ^+^ CD8^+^ T cells was determined. Representative dot plots for the IFN-γ synthesis in gated CD8^+^ T cells are shown. Numbers in the regions indicate the percentage of positive cells. **(I)** Purified splenic CD8^+^ T cells were exposed to various concentrations of P3CSK4. After 18 h, the cells were stimulated with IL-12/IL-15/IL-18, and the content of IFN-γ in the supernatant was determined. Data show mean ± SD of triplicate cultures and are representative for three independent experiments. Statistical differences versus P3CSK4 (0 µg/ml) were tested using unpaired Student t-test. *p ≤ 0.05; **p ≤ 0.01; ***p ≤ 0.001.

Moreover, we evaluated the synthesis of IFN-γ by CD8^+^ T cells from WT and TLR2^−/−^ mice after CLP. Thereby, stimulation with PMA/Ionomycin or with a cytokine cocktail was used to mimic complete T-cell activation by TCR signaling and costimulation (36) and antigen-independent activation, respectively. In WT BM, CD8^+^ T cells displayed a reduced capacity to produce IFN-γ after CLP, whereas CD8^+^ T cells in TLR2^−/−^ BM preserved their IFN-γ synthesis (**Figures 8A, B**). Impaired IFN-γ synthesis in T cells is often associated with increased programmed cell death protein (PD)-1 expression, indicating T cell exhaustion (37). Because T cell exhaustion is considered to develop during sepsis (14), we determined the expression of PD-1 on CD8^+^ T cells. Unexpectedly, CD8^+^ T cells in the BM of WT mice decreased their expression of PD-1 during sepsis, whereas the expression of PD-1 remained unchanged in TLR2^−/−^ mice (**Figure 8C**).

Because TLR2 is expressed on numerous cell types, it remained unclear whether the reduced synthesis of IFN-γ by CD8^+^ T cells after CLP mirrored a direct or indirect effect of TLR2 ligands on CD8^+^ T cells. To address this issue, we generated a model in which the behavior of WT versus TLR2^−/−^ CD8^+^ T cells could be examined in the same microenvironment of WT BM. Therefore, purified CD8^+^ T cells from WT or TLR2^−/−^ mice were adoptively transferred into congenic CD45.1^+^ WT mice before induction of sepsis. In this experimental setting, endogenous and transferred CD8^+^ T cells in the BM showed the same characteristics (i.e., increased number of T_N_, T_CM_, and T_VM_ cells and elevated expression of CD69; **Supplementary Figure 9**), as previously observed for CD8^+^ T cells in WT and TLR2^−/−^ mice. The transferred CD8^+^ T cells from WT or TLR2^−/−^ mice accounted for 10% of total CD8^+^ T cells in the BM of both sham and CLP mice (**Figure 8D**). The comparison with the original subset distribution of donor cells before administration into the recipient revealed that predominantly T_Eff/EM_, T_CM_, and T_VM_ cells sequestered in the BM of sham and CLP mice (**Supplementary Figure 10**). Moreover, the subset distribution did not differ between transferred cells from WT and TLR2^−/−^ mice (**Supplementary Figure 10**).

Endogenous CD45.1^+^ CD8^+^ T cells and transferred CD45.1^−^ CD8^+^ T cells from WT mice displayed a decreased IFN-γ production after CLP. In contrast, transferred CD8^+^ T cells from TLR2^−/−^ mice did not change their IFN-γ synthesis although they were surrounded by WT BM (**Figure 8E**). Gene expression profiling of BMCs in the same adoptive transfer setting indicated increased gene expression related to the IFN-γ response pathway after transfer of CD8^+^ T cells from TLR2^−/−^ mice in comparison with the transfer of WT cells (**Figures 8F, G** and **Supplementary Figure 11**).

The finding that CD8^+^ T cells from TLR2^−/−^ mice did not show a decline in IFN-γ synthesis after CLP implied that under certain circumstances TLR2 agonists may suppress the activity of CD8^+^ T cells. To investigate this hypothesis, BMCs from naïve WT mice were exposed to the TLR2 agonist P3CSK4 *in vitro* and were stimulated with PMA/Ionomycin 18 h later. Pre-exposure to P3CSK4 caused a reduced IFN-γ synthesis in CD8^+^ T cells (**Figure 8H**). The inhibitory effect of P3CSK4 on the IFN-γ production was confirmed with purified splenic CD8^+^ T cells and cytokine (IL-12/IL-15/IL-18)–induced stimulation of the cells (**Figure 8I**). Thus, agonists of TLR2 are responsible for the reduced IFN-γ synthesis of CD8^+^ T cells in the BM during sepsis.

### The presence of IFN-γ during differentiation shapes the response of DCs to TLR ligands

The transfer of TLR2^−/−^ CD8^+^ T cells changed the local environment in the BM after CLP and thereby might affect the differentiation of DCs. To address this assumption, CD8^+^ T cells from WT or TLR2^−/−^ mice were adoptively transferred into WT mice before CLP as described above. After 4 days, the BM and spleen cells were isolated. The transfer of TLR2^−/−^ CD8^+^ T cells did not change the number or subset distribution of pre-DCs in the BM nor of conventional DCs in the spleen (**Supplementary Figure 12**).

**Figure 9:**
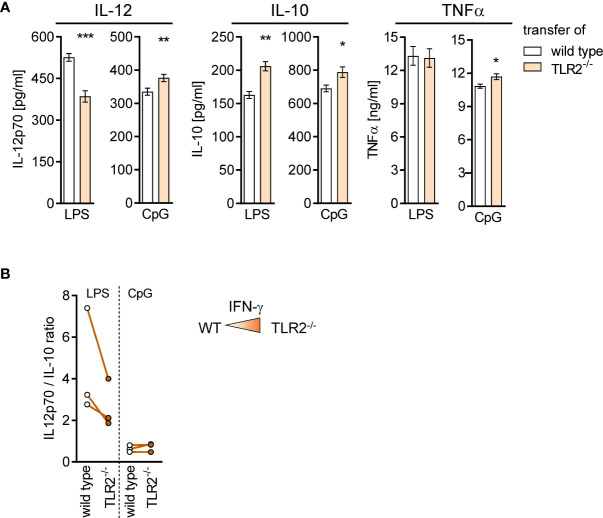
TLR2^−/−^ CD8^+^ T cells shape the function of differentiating DCs in the BM during sepsis. Sepsis was induced in WT mice after adoptive transfer of WT or TLR2^−/−^ CD8^+^ T cells as described in **Figure 8**. After 4 days, BM cells were isolated and BM-derived DCs (BMDCs) were generated *in vitro*. **(A)** Release of IL-12p70, IL-10, and TNFα after stimulation of BMDC with LPS or CpG. Data show mean ± SD of triplicate cultures from one representative experiment. Unpaired t-test was performed for statistical analysis. **(B)** Ratio of IL-12/IL-10 secretion of BMDC from three independent experiments. As a reminder, the color intensity in the triangle illustrates the increased IFN-γ signaling in the BM after transfer of TLR2^−/−^ CD8^+^ T cells. *p ≤ 0.05; **p ≤ 0.01, ***p ≤ 0.001.

BMDC generated from BM of mice after transfer of TLR2^−/−^ CD8^+^ T cells secreted less IL-12 but more IL-10 upon stimulation with LPS and accordingly displayed a decreased ratio of IL-12/IL-10 (**Figures 9A, B**). No change in the ratio of IL-12/IL-10 between cells of both groups was observed after stimulation with CpG (**Figure 9B**). Thus, TLR2^−/−^ CD8^+^ T cells modulate the microenvironment in the BM and influence the cytokine secretion of differentiating DCs.

**Figure 10:**
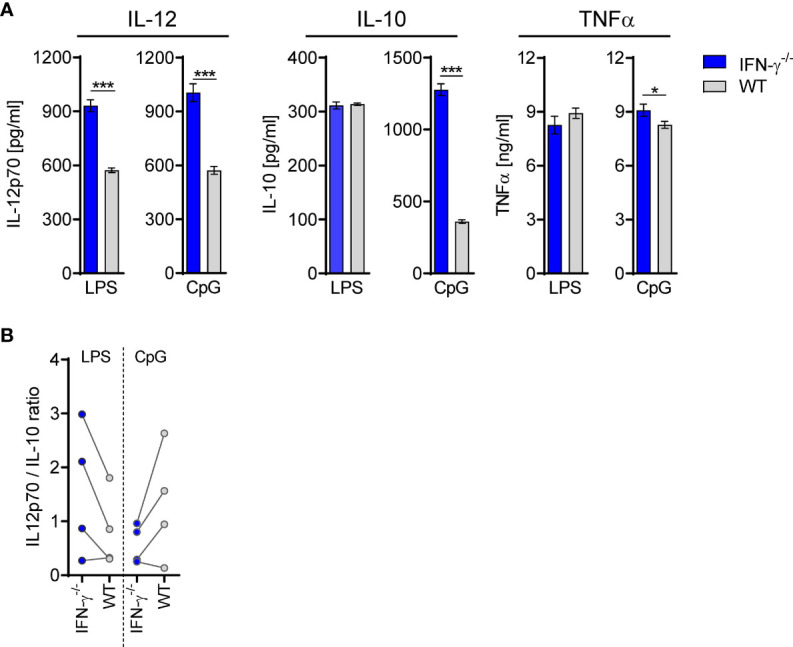
DCs from IFN-γ^−/−^ mice secrete enhanced levels of IL-12. BMDCs were generated from WT or IFN-γ^−/−^ mice and stimulated with LPS or CpG. **(A)** Release of IL-12p70, IL-10, and TNFα. Data show mean ± SD of triplicate cultures from one representative experiment. Unpaired t-test was performed for statistical analysis. **(B)** Ratio of IL-12/IL-10 secretion of BMDC from four independent experiments. *p ≤ 0.05; ***p ≤ 0.001.

Taking into consideration that the altered cytokine secretion of BMDC after transfer of TLR2^−/−^ CD8^+^ T cells was associated with enhanced IFN-γ signaling in the BM, we asked whether IFN-γ, when present during differentiation, in general, causes an altered cytokine secretion profile of DCs. Therefore, the cytokine secretion of BMDC generated from IFN-γ^−/−^ and WT mice was investigated. Increased secretion of IL-12 but not IL-10 was observed for IFN-γ^−/−^ BMDC upon stimulation with LPS that led to an increased ratio of IL-12/IL-10 (**Figures 10A, B**). Vice versa, the presence of recombinant IFN-γ during generation of BMDC from IFN-γ^−/−^ mice led to a decreased IL-12 secretion upon stimulation with LPS (**Supplementary Figure 13A**) and to a reduced ratio of IL-12/IL-10 (**Supplementary Figure 13B**). In contrast, IFN-γ^−/−^ BMDC displayed a decreased ratio of IL-12/IL-10 in comparison with WT BMDC when stimulated with CpG (**Figures 10A, B**), which was not affected by the presence of recombinant IFN-γ (**Supplementary Figure 13B**). Thus, the presence of IFN-γ during differentiation shapes the cytokine secretion of DCs in response to TLR ligands.

## Discussion

Nosocomial infections represent a major cause of death of patients surviving the initial hyperinflammation during sepsis. An altered differentiation of DCs in the BM, T-cell lymphopenia, and a compromised synthesis of IFN-γ by T cells are considered to contribute to the impaired immune defense during sepsis although the underlying mechanisms are less clear. In this study, we discovered that CD8^+^ T cells are required to restore the number and function of differentiating DCs in the BM during sepsis. We identified cell-intrinsic signaling through TLR2 as the origin of impaired IFN-γ synthesis by CD8^+^ T cells during sepsis that unexpectedly limited the dysregulation of differentiating DCs in the BM.

Our initial characterization of the T cell compartment revealed a notable increase of the CD4^+^ and CD8^+^ T cell number in the BM early after induction of sepsis. This novel finding is in contrast to the decades-old paradigm of a generalized apoptosis-induced lymphopenia in blood, lymphoid, and non-lymphoid tissues (38). Among CD8^+^ T cells, mainly the T_N_, T_CM_, and T_VM_ subpopulations accumulated in the BM during sepsis. We did not find evidence for an increased proliferation of CD8 T cells in septic mice that might have explained the expansion of these T cell subsets after CLP. Because adoptively transferred T cells that appeared in the BM displayed a similar subset distribution as did endogenous T cells, we assume that sepsis induced a selective recruitment of T_N_, T_CM_, and T_VM_ CD8^+^ T cells from the circulation into the BM.

In homeostasis, the BM provides high levels of CXCL12 that mediates retention of naïve and memory CD8^+^ T cells after their entry from the circulation (39). Limited information exists on the migratory behavior of T cell subpopulations during sepsis: CXCR4, the receptor for CXCL12, is expressed on naïve and memory T-cell subsets early during sepsis, and the pharmacological inhibition of CXCR4 prevents lymphopenia in the spleen, whereas it increases the number of T cells in the blood (40). These findings point to a redistribution of T cells between different compartments during sepsis. Consistent with a previous report (41), we observed an increased expression of CD69 on memory CD8^+^ T cells in the BM as well as in the spleen early during sepsis indicating a systemic activation of CD8^+^ T cells. CD69 interferes with the sphingosin-1-phosphate–mediated exit from tissues into the circulation (42, 43). Thus, CXCR4 and CD69 might contribute to the selective recruitment and retention of CD8^+^ T_N_, T_CM_, and T_VM_ cells in the BM during sepsis, a hypothesis that remains to be evaluated further.

CD8^+^ T_VM_ cells were the subpopulation among CD8^+^ T cells that displayed the activated phenotype at highest frequency. CD8^+^ T_VM_ cells are superior in antigen-independent or “bystander” activation than other CD4^+^ and CD8^+^ T cell subsets presumably due to their expression of the IL-15 receptor β chain (44). Adoptively transferred TCR transgenic CD8^+^ T_VM_ cells from DO11.10 mice increased their expression of CD69 after CLP to a similar degree as did the transferred WT counterparts. This finding supports the assumption that the activation of CD8^+^ T_VM_ cells occurred independent from their cognate antigen. Bystander CD8^+^ T cell activation is driven by diverse innate cytokines such as IL-12, IL-18, IFN-α, IL-2, or IL-15 (7) that all have been detected in the septic host (45). The identification of the signals that drive bystander activation of CD8^+^ T_VM_ cell during sepsis and the relevance of disease severity herein remain to be addressed in future work.

In line with earlier studies, we observed a fulminant decline in the number of DC1 and DC2 in the spleen during sepsis (23–25) that is mediated at least in part by apoptosis-induced cell death (46, 47). Here, we provide first evidence that the reduction in the number of splenic DCs is associated with a profound contraction of the pre-DC population in the BM within the first 24 h after CLP. A reduced number or frequency of DC progenitor cells has also been observed in patients with pediatric sepsis and in murine models of systemic infections with bacteria such as *Yersinia pestis* or with *Influenza A virus* (48–50). The decline in DC progenitor cells is explained by an increased mobilization of pre-DCs from BM to replenish the DC population in peripheral tissues or by a shift toward increased monopoiesis at the expense of DC progenitors (49, 51). Whether any of these mechanisms likewise applies to sepsis and to which extent the low number of pre-DCs is responsible for the sustained reduction of the DC population in the periphery is currently under investigation.

Importantly, we observed that the pre-DC population in the BM recovered by 4 days after induction of sepsis, which was dependent on the presence of CD8^+^ T cells. This finding points to a so far unrecognized function of CD8^+^ T cells during sepsis that is distinct to their classical role in the elimination of virus-infected cells or tumor cells. Because the number of CD8^+^ T cells declined beyond 24 h after CLP, we assume that CD8^+^ T cells acted on yet undefined stages of DC progenitors early during sepsis. The elucidation of the molecular mechanisms underlying the impact of CD8^+^ T cells on DC progenitors during sepsis requires more detailed analyses of T cells in terms of localization and expression of growth factors in the BM.

CD8^+^ T cells did not only support the rise in the pre-DC number but moreover shaped the function of DCs as they favored the differentiation of DCs toward increased IL-12 synthesis during sepsis. This finding strengthens the concept that the cytokine secretion pattern of DCs relies not only on the local tissue environment in the periphery (19, 22) but also on signals that they incorporated during earlier differentiation stages in the BM. Given that immediate DC precursors are released from BM and colonize diverse tissues throughout the body, any modulation of DC progenitor cells in the BM will result in a systemic alteration of the DC compartment. Accordingly, lymphopenia that persists for several weeks after onset of sepsis (52) might interfere with DC differentiation and thereby might contribute to the chronic dysregulation of DC function (30, 53). In our opinion, this is a novel function of CD8^+^ T cells during sepsis that is independent from their cognate antigen and might increase the susceptibility to opportunistic pathogens that do not rely on specific T-cell immunity for efficient elimination.

Data on the altered T cell function during sepsis largely rely on human circulating and murine splenic T cells (54). T cell exhaustion is regarded as the mechanism underlying diminished IFN-γ synthesis by T cells during sepsis (55, 56). Splenic T cells express the characteristic exhaustion marker PD-1 from 48 h after CLP (14). We observed that, by 24 h after CLP, CD8^+^ T cells were impaired in IFN-γ synthesis but did not increase the expression of PD-1. Therefore, exhaustion does not explain the early decline in IFN-γ production of CD8^+^ T cells in the BM during sepsis. We discovered that the deficit in IFN-γ synthesis was rather the direct consequence of prior signaling through TLR2 in CD8^+^ T cells.

TLR2 is well known as a pattern recognition receptor on innate immune cells (57). Less appreciated is the fact that TLR2 is also expressed on T lymphocytes where it acts as a co-stimulatory molecule and synergizes with TCR-driven T cell activation (34). In contrast, we noticed that prior exposure of CD8^+^ T cells to the TLR2 ligand P3CSK4 led to a diminished IFN-γ synthesis by CD8^+^ T cells and, thus, mirrored the modulation of CD8^+^ T cells in the BM during sepsis. Therefore, we suggest that TLR2 ligands that may derive from intestinal bacteria or belong to the group of alarmins (58) are responsible for the impaired IFN-γ production of CD8^+^ T cells early during sepsis. The mechanisms underlying the TLR2-mediated deactivation of CD8^+^ T cells are currently under investigation.

The adoptive transfer experiments allowed us to selectively study the impact of TLR2-expressing CD8^+^ T cells on the WT BM environment in the septic host. The superior expression of IFN-γ in CD8^+^ T cells from TLR2^−/−^ mice was associated with enhanced gene expression of the IFN-γ response pathway in the BM. Given that the adoptively transferred CD8^+^ T cells accounted for less than 0.2% of total BMCs, this finding points to a strong impact of TLR2^−/−^ CD8^+^ T cells on the BM microenvironment. Indeed, BMCs that harbored transferred TLR2^−/−^ CD8^+^ T cells gave rise to BMDC, which displayed a reduced IL-12/IL-10 ratio. This finding suggests that IFN-γ in the BM promotes the differentiation of DCs with a less immune-activating potential that is supported by the fact that BMDC from IFN-γ^−/−^ mice displayed an increased IL-12/IL-10 ratio in line with a previous study (59). In this context, it might appear contradictory that BMDC generated after depletion of CD8^+^ T cells that represent a source of IFN-γ did not behave like BMDC from IFN-γ^−/−^ mice. Most likely, CD8^+^ T cells do not only release IFN-γ but also diverse mediators that favor the differentiation of immune-activating BMDC and outweigh the effect of IFN-γ as driver of regulatory BMDC. The identification of such relevant mediators of CD8^+^ T cells in the BM is subject of current and future work.

The direct immunostimulatory activity of IFN-γ on DCs and monocytes/macrophages confers protection from diverse primary microbial infections and is well described (60). In contrast, a recent report from Kim et al. showed that endogenous IFN-γ impairs the function of macrophages during sepsis and increases the susceptibility to secondary infection (61). In addition, our data reveal an indirect and rather long-term effect of IFN-γ on DC function as it instructs progenitor cells to differentiate to DCs with restricted IL-12 synthesis. From the opposite point of view, the decreased IFN-γ production by CD8^+^ T cells that develops during sepsis favors the differentiation of IL-12–secreting DCs. Thus, IFN-γ may exert both stimulatory and regulatory activities, depending on the current immune state of the host (62). Consequently, attempts to restore the IFN-γ production of T cells or the therapeutic administration of recombinant IFN-γ during sepsis harbor the risk to aggravate the dysregulation of DCs and presumably other immune cells through long-lasting modulation of the BM microenvironment (63). Therefore, we propose to revisit the use of IFN-γ as immune-stimulatory therapy during sepsis.

In summary, early during polymicrobial sepsis, bystander-activated CD8^+^ T cells accumulate in the BM and support the differentiation of pre-DCs. In parallel, CD8^+^ T cells lose their capacity to produce IFN-γ upon signaling through TLR2 that limits the development of DC dysregulation. Therapeutic approaches that increase the number of CD8^+^ T cells without amplifying T cell–derived IFN-γ synthesis might support the restoration of DC function during sepsis.

## Data availability statement

The data presented in the study are deposited in the GEO repository, accession number: GSE210981.

## Ethics statement

This study was reviewed and approved by Local ethic committee Landesamt für Natur-, Umwelt-, und Verbraucherschutz (LANUV), North-Rhine-Westphalia.

## Author contributions

A-CA, EP, JJ, LK-H, and SBF designed, performed experiments and/or analyzed data. A-CA and SBF wrote the manuscript, and all authors made editorial suggestions and approved the final version. All authors contributed to the article and approved the submitted version.

## Funding

The study was supported by the Deutsche Forschungsgemeinschaft DFG (GRK1949 to SBF).

## Acknowledgments

We are grateful to Michaela Bak, Marion Frisch, and Nadine Gausmann for excellent technical assistance. We thank Lisa Wienhöfer for her valuable support in the animal work and flow cytometry.

## Conflict of interest

The authors declare that the research was conducted in the absence of any commercial or financial relationships that could be construed as a potential conflict of interest.

## Publisher’s note

All claims expressed in this article are solely those of the authors and do not necessarily represent those of their affiliated organizations, or those of the publisher, the editors and the reviewers. Any product that may be evaluated in this article, or claim that may be made by its manufacturer, is not guaranteed or endorsed by the publisher.
