# Supplementary material for: TLR2-induced CD8+ T-cell deactivation shapes dendritic cell differentiation in the bone marrow during sepsis
Source: Front Immunol. 2022 Sep 6;13:945409. doi: 10.3389/fimmu.2022.945409 (PMC9488929; doi:10.3389/fimmu.2022.945409)
Supplement: Supplementary file 1 [file DataSheet_1.pdf]

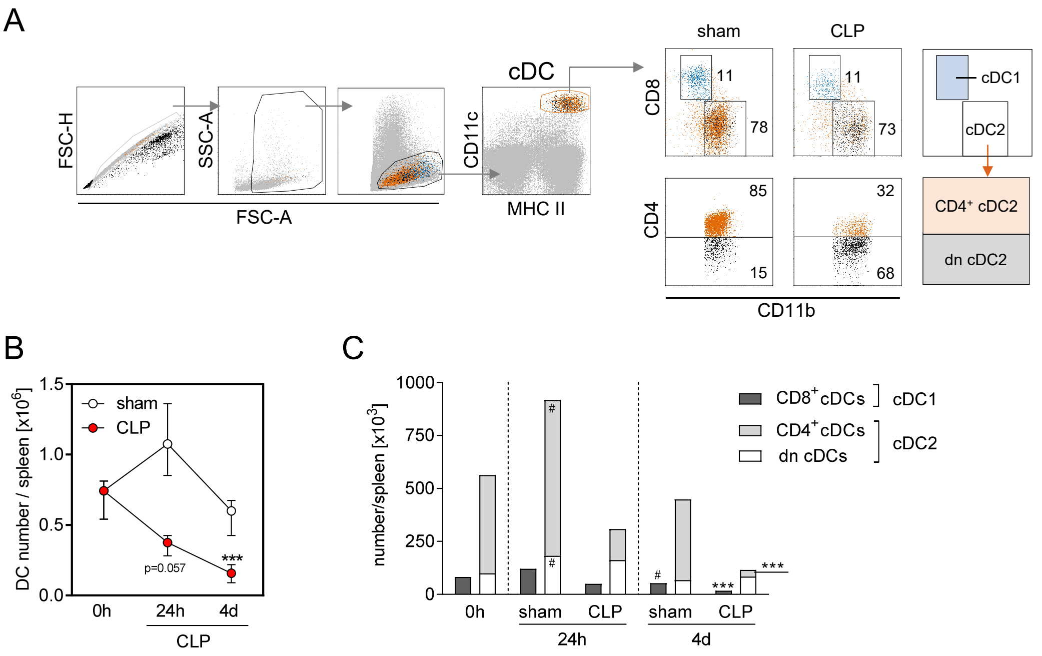

Supplementary Figure 1. Loss of $CD4^+$ cDC2 during the course of sepsis. Total spleen cells were isolated at the indicated time points after CLP or sham surgery of wild type mice and cDC subsets were analyzed by flow cytometry. (A) Gating strategy of $CD11c^{hi}$ MHC II^+ cDCs. Representative dot plots of one sham and one CLP mouse (4d after surgery) are shown. cDC subtypes were identified as $CD8^+CD4^-CD11b^-$ cDC1 and $CD8^-CD11b^+$ cDC2. The latter were further separated into $CD4^+$ cDC2 and $CD8^-CD4^-$ (dn) cDC2. Numbers indicate the percentage of positive cells. (B) Absolute number of cDCs per spleen. Data show the median and interquartile range. (C) Distribution of cDCs on cDC1 and cDC2 subsets. Data show the median. Significant differences were tested using the Mann-Whitney U-test. 0h and 24h: n=3-4 mice/group; 4d n=10-14 mice per group. *, $p \leq 0.05$; **, $p \leq 0.01$; ***, $p \leq 0.001$ sham versus CLP. #, $p \leq 0.05$ sham versus 0h.

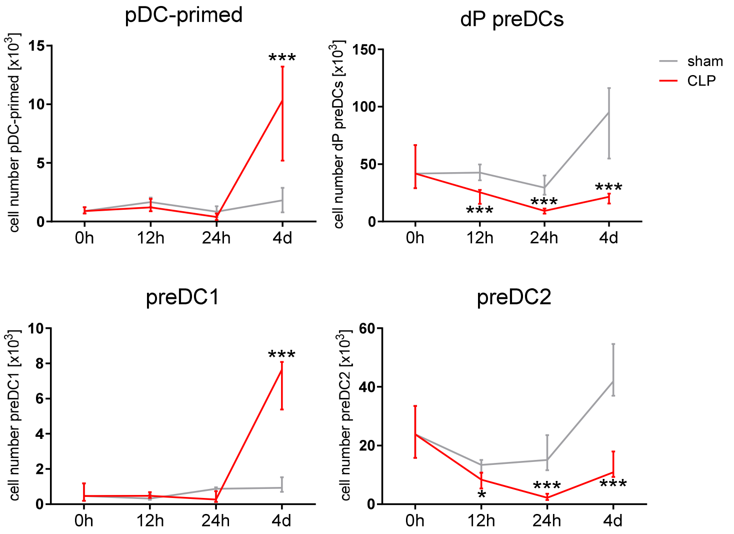

Supplementary Figure 2. Kinetics of the absolute number of pre-DC subsets in the bone marrows during sepsis. At different time points after CLP or sham surgery, the absolute number of each preDC subset was determined. Values indicate the number per mouse**.** Data show the median with interquartile range of n=4 (0h; equivalent to naïve mice) or n=7-14 mice per group. Significant differences were tested using Mann-Whitney *U*-test. *, $p \leq 0.05$; ***, $p \leq 0.001$ dP, double positive; pDC, plasmacytoid DC

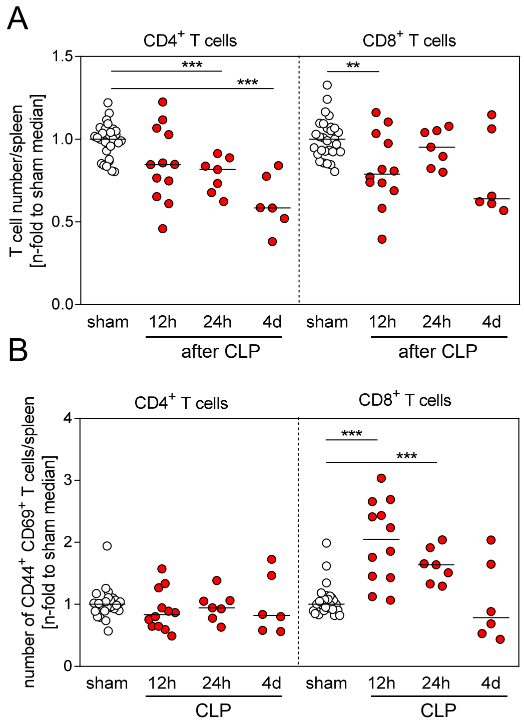

Supplementary Figure 3. Number and activation of splenic T cells after CLP. Sepsis was induced in wildtype mice and total spleen cells were isolated at the indicated time points after CLP or sham surgery. T cells were analyzed by flow cytometry according to the gating strategy given in Figure 3. The T cell number (A) and the number of $CD69^{+}CD44^{+}$ T cells (B) per spleen were normalized to the median of the respective sham values. Non-parametric Mann-Whitney *U*-test was performed for statistical analysis. *, $p \leq 0.05$; **, $p \leq 0.01$; ***, $p \leq 0.001$

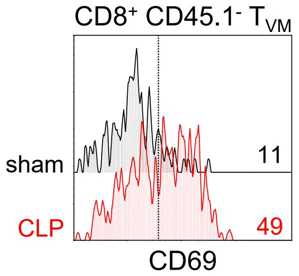

Supplementary Figure 4. Increased expression of CD69 on transferred wildtype $CD8^+$ T_{VM} cells in the bone marrow after CLP. $CD8^+$ T cells from WT mice were adoptively transferred into $CD45.1^+$ congenic mice prior to CLP or sham surgery. After 24 h, transferred cells in the bone marrow were identified as $CD45.1^-$ cells and the expression of CD69 on $CD8^+$ T_{VM} cells was evaluated. The histogram depicts the expression of CD69 on gated $CD45.1^-$ $CD8^+$ T_{VM} cells from one representative sham and one CLP mouse. T_{VM}, virtual memory T cell

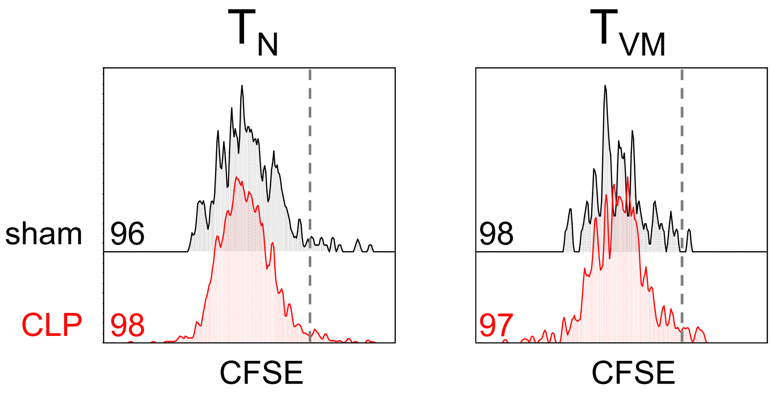

Supplementary Figure 5. Proliferative activity of CD8$^+$ T cells in the bone marrow after CLP. CFSE-labeled splenic CD8$^+$ T cells were adoptively transferred immediately after sham or CLP surgery. After 24 h, bone marrow cells were isolated and the content of CFSE in gated CD8$^+$ T_N and T_{VM} cells was determined by flow cytometry. Histograms show the CFSE fluorescence intensities each from one representative sham and CLP mouse. The dashed line separates undivided cells from cells that underwent at least one division. Numbers indicate the percentage of divided cells.

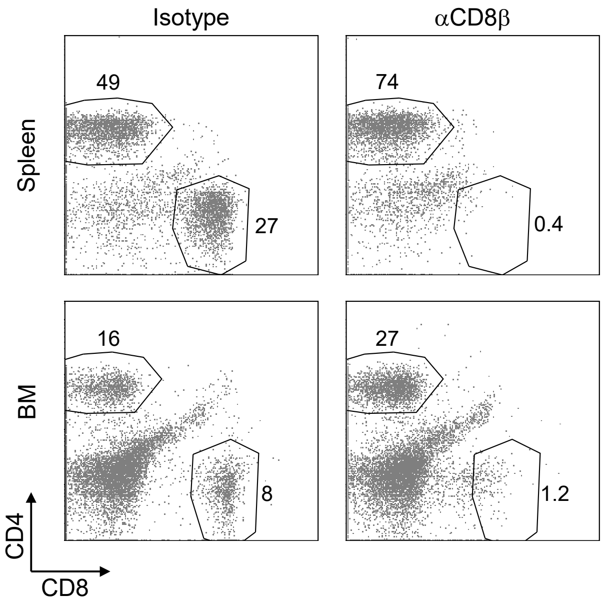

Supplementary Figure 6. Efficiency of CD8$^+$ T cell depletion in bone marrow and spleen. Sepsis was induced in wildtype mice after CD8$^+$ T cell depletion or isotype application. The dot plots show the composition of CD4$^+$ and CD8$^+$ T cells in bone marrow (BM) and spleen 4 days after CLP. Numbers indicate the percentage share after gating for CD3$^+$ T cells.

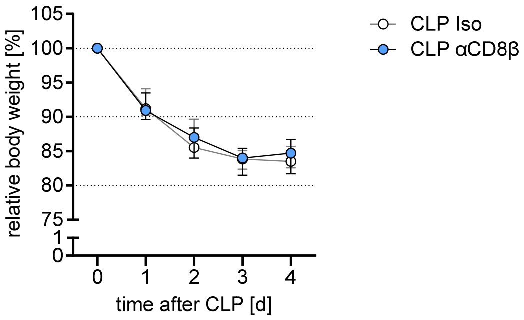

Supplementary Figure 7. Depletion of CD8 T cells does not affect sepsis-induced body weight loss. Sepsis was induced in wildtype mice after the application of αCD8β (n=5) or isotype control (n=8) antibodies. The body weight was monitored before (0 d) and until 4 d after CLP and normalized to the value on d0 (set as 100%). Data show median/interquartile range.

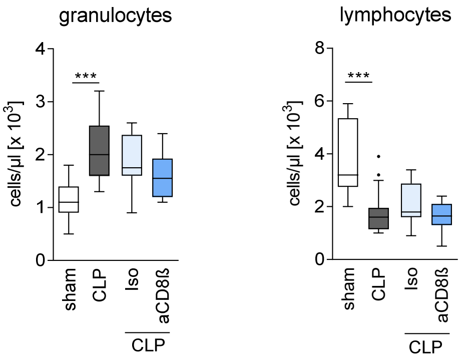

Supplementary Figure 8. Number of granulocytes and lymphocytes in the blood 4 d after sepsis. Wildtype mice underwent sham or CLP surgery. Two groups of CLP mice received depleting antibodies against CD8β (aC8ß) or the respective isotype control antibody (Iso) before induction of sepsis. The number of granulocytes and lymphocytes in the blood was quantified 4 d later by Vet abc™ . The results are shown as Tukey box plots of n=13-17 mice per group. Nonparametric Mann-Whitney *U*-test was used for statistical analysis. ***, $p \leq 0.001$

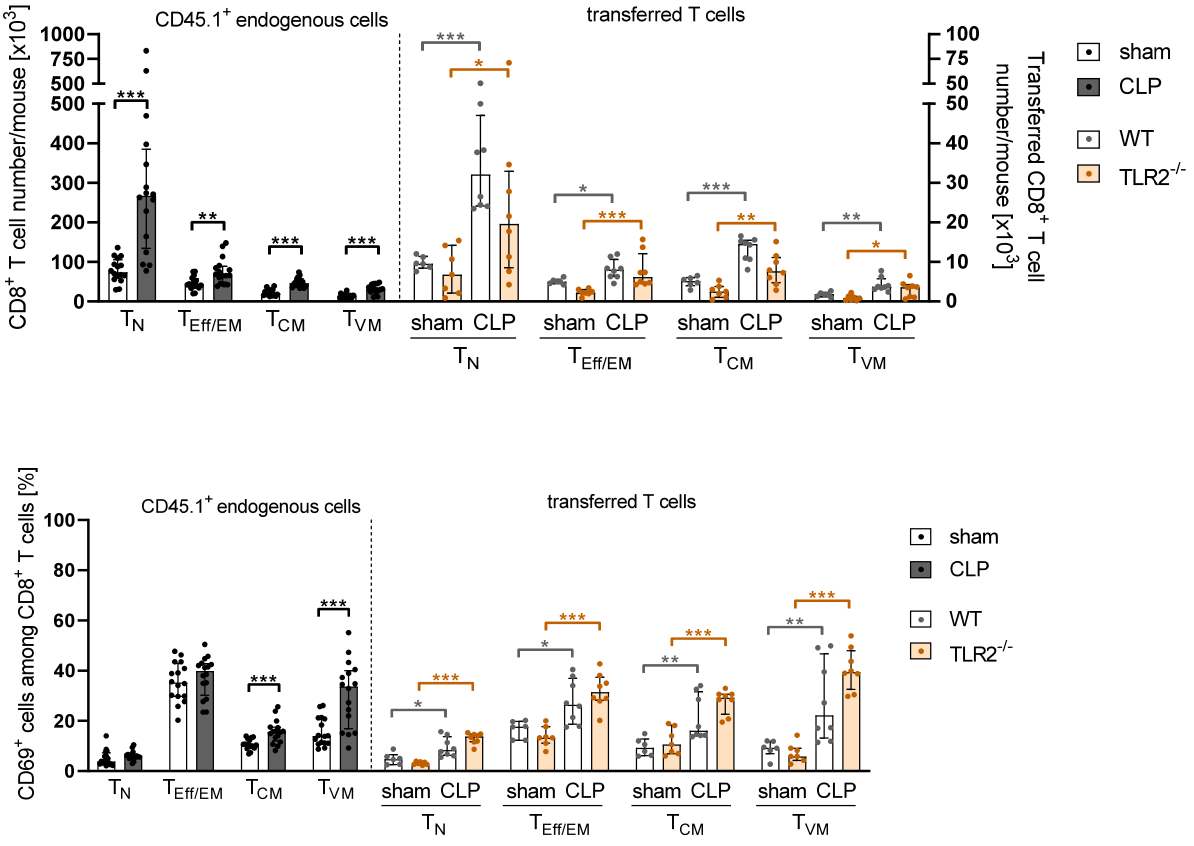

Supplementary Figure 9. Number of endogenous and transferred CD8$^+$ T cells and expression of CD69 in the bone marrow. CD45.1$^+$ congenic mice received CD8$^+$ T cells from WT (CD45.1$^-$) or from TLR2$^{-/-}$ (CD45.1$^-$) mice before CLP or sham surgery and BM cells were isolated 24 h later (n=7-8 mice per group). Total CD8$^+$ T cells were gated and endogenous (endo) CD45.1$^+$ and transferred CD45.1$^-$ CD8$^+$ T cells were discriminated. (A) Absolute number of T cells of distinct T cell subsets. (B) Frequency of CD69$^+$ cells among distinct T cell subsets. Bar graphs show median and interquartile range as well as individual values. Statistical differences were tested using Mann-Whitney U-test. *, $p \leq 0.05$; **, $p \leq 0.01$; ***, $p \leq 0.001$. T_N, naïve; $T_{Eff/EM}$, effector/effector memory; T_{CM}, central memory; T_{VM}, virtual memory CD8$^+$ T cells

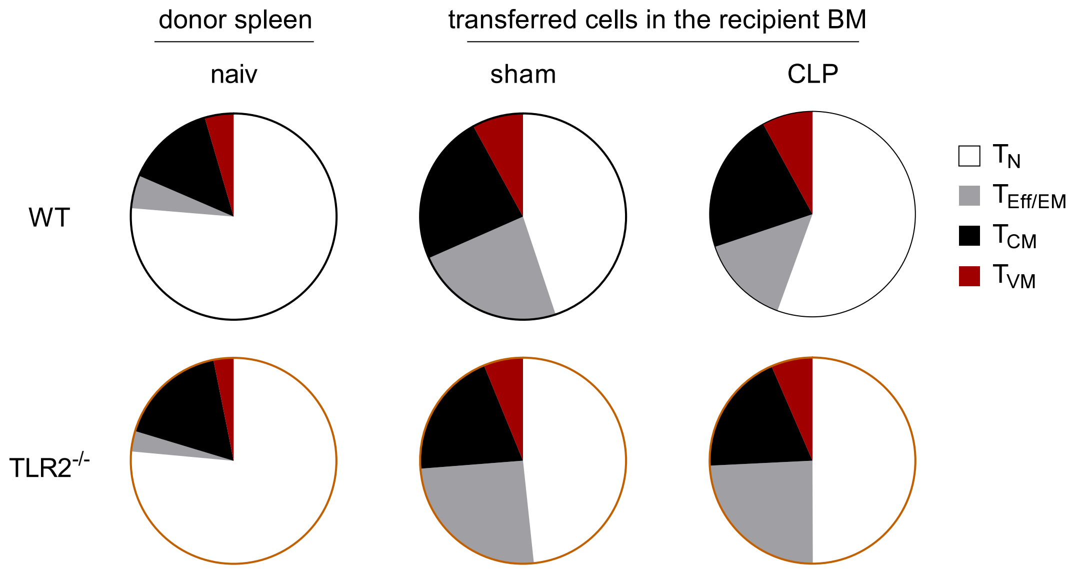

Supplementary Figure 10. Distribution of splenic CD8^{+} T cell subpopulations from wildtype and TLR2$^{-/-}$ mice before and after transfer. Pie charts show the subtype distribution of CD8^{+} T cells in the naïve donor spleen of wildtype (n = 5) or TLR2$^{-/-}$ mice (n = 3) and after their appearance in the bone marrow (BM) of recipient sham and CLP mice (n = 7-8). Pie chart values represent the median. T_{N}, naïve; $T_{Eff/EM}$, effector/effector memory; T_{CM}, central memory; T_{VM}, virtual memory

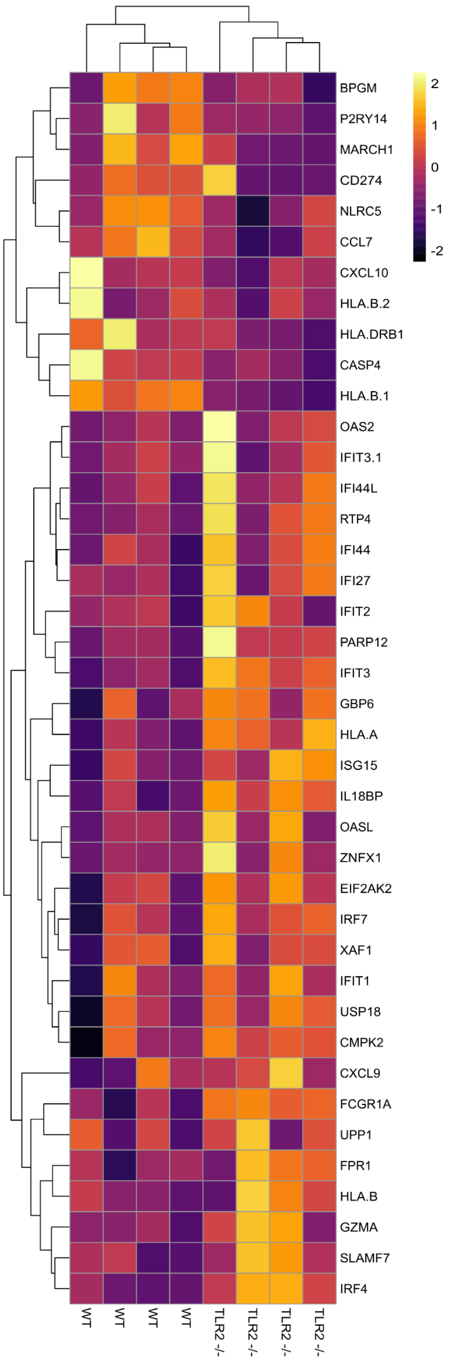

Supplementary Figure 11. Heatmap of the top 40 differentially expressed genes in the Interferon gamma response pathway (Hallmark) in the bone marrow of septic mice after transfer of wildtype or TLR2$^{-/-}$ CD8^{+} T cells. Genes shown have at least an absolute log2 difference of 0.2. Heatmap was created with the R-package pheatmap (v1.0.12, Raivo Kolde, 2019). Samples as well as genes were clustered with euclidean distance values. Color values shown are scaled within each row.

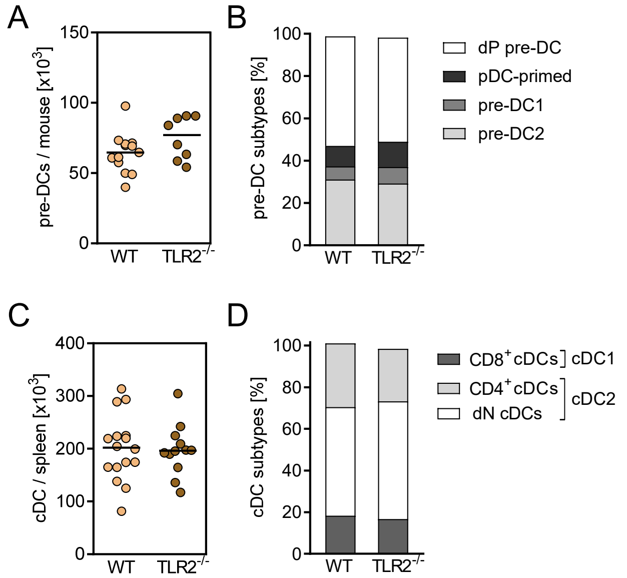

Supplementary Figure 12. CD8^{+} T cells from TLR2$^{-/-}$ mice do not influence the number of pre-DCs and cDCs. Sepsis was induced in wild type mice after transfer of wild type (WT) or TLR2$^{-/-}$ CD8^{+} T cells (TLR2$^{-/-}$). After 4d, pre-DCs in the bone marrow and cDC in the spleen were analyzed by flow cytometry. The number (A, C) and composition (B, D) of pre-DCs (A, B) and cDCs (C, D) are shown as the median of n=8-16 mice per group. Non-parametric Mann-Whitney U-test was performed for statistical analysis.

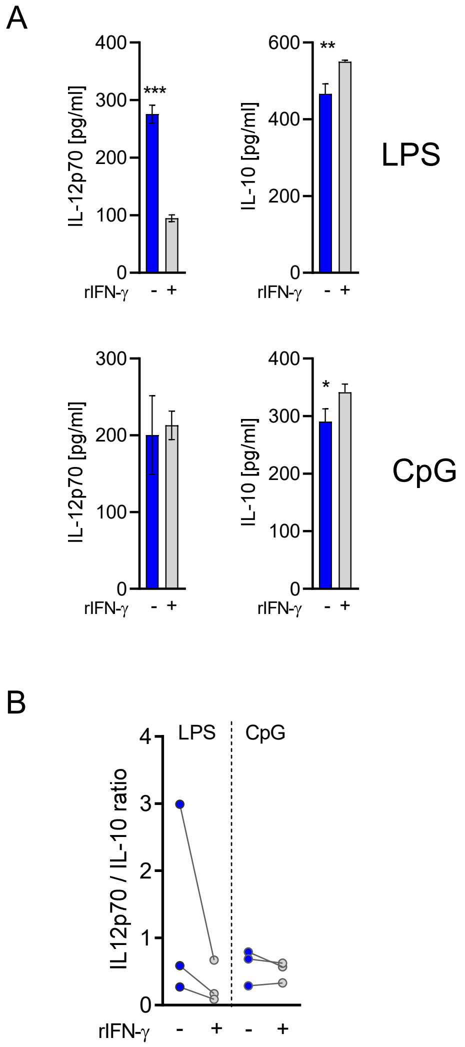

Supplementary Figure 13. The presence of recombinant IFN-γ during the generation of BMDC from IFN-γ$^{-/-}$ mice decreases the ratio of IL-12/IL-10 cytokine secretion. BMDC were generated from IFN-γ$^{-/-}$ mice in the presence or absence of 0.1 ng/ml recombinant murine IFN-γ and stimulated with LPS or CpG. (A) Release of IL-12p70 and IL-10. Data show mean±SD of triplicate cultures from one representative experiment. Unpaired t-test was performed for statistical analysis. (B) Ratio of IL-12/IL-10 secretion of BMDC from three independent experiments. *, $p \leq 0.05$; **, $p \leq 0.01$; ***, $p \leq 0.001$
